# Supplementary material for: Content-rich biological network constructed by mining PubMed abstracts
Source: BMC Bioinformatics. 2004 Oct 8;5:147. doi: 10.1186/1471-2105-5-147 (PMC528731; doi:10.1186/1471-2105-5-147)
Supplement: Additional File 2 — The original results of the above study (non-essential files are deleted to keep the file size under the limit set by BMC bioinformatics). [file 1471-2105-5-147-S2.bz2 › chilibotAdditionalFile2/dip05/236ID8006584E760/html/IL2RG_IL2.html]

 


 **IL2RG** and **IL2** 
  
Found 241 abstracts in PubMed, retrieved 05.  
 

 What does Google say? 
 PDF only 
| .edu only 

---

**Interactive relationship** (e.g. stimulation, inhibition, etc)

**Neutral relationship**- The receptor consists of two chains IL 7 specific alpha chain CD127 and the common gamma c  [ **IL2RG** ]  chain CD132  [ **IL2RG** ]  which is a component of several other cytokine receptors IL 2  [ **IL2** ] , IL 4, IL 9 and IL 15.  Ref: 12644318 Immunol Lett, 2003
- Our laboratory has identified an X linked severe combined immunodeficiency XSCID  [ **IL2RG** ]  in dogs that is the result of mutations in the common gamma chain gammac subunit of the interleukin 2  [ **IL2** ]  IL 2  [ **IL2** ] , IL 4, IL 7, IL 9, IL 15, and IL 21 receptors.  Ref: 12857971 Immunol Res, 2003

**Non-interactive relationship** (e.g. studied together, co-existance, homology, etc.)

- The chicken B cell marker ChB6, caspase 1, inhibitor of apoptosis protein 1 IAP 1, interleukin 15 receptor alpha chain IL 15Ralpha, interleukin 2  [ **IL2** ]  receptor gamma chain  [ **IL2RG** ]  IL 2Rgamma, and immunoglobulin supfamily gene ZOV3, as physiological candidate genes for chicken immune response, were selected to investigate associations with antibody kinetics to SRBC and killed B. abortus.  Ref: 12872968 Poult Sci, 2003
- SOCS 1 regulates signaling in response to IL 2  [ **IL2** ]  and other gamma c  [ **IL2RG** ]  dependent cytokines in peripheral T cells.  Ref: 12665516 J Biol Chem< MedlineTA>J Biol Chem, 2003
